# Capturing Subjective Mild Cognitive Decline in Parkinson’s Disease

**DOI:** 10.3390/brainsci12060741

**Published:** 2022-06-05

**Authors:** Sara Rosenblum, Sonya Meyer, Ariella Richardson, Sharon Hassin-Baer

**Affiliations:** 1 Laboratory of Complex Human Activity and Participation (CHAP), Department of Occupational Therapy, Faculty of Social Welfare & Health Sciences, University of Haifa, Haifa 3498838, Israel; 2 Department of Occupational Therapy, Ariel University, Ariel 4077603, Israel; sonyam@ariel.ac.il; 3 Department of Industrial Engineering, Jerusalem College of Technology, Jerusalem 9372115, Israel; richards@jct.ac.il; 4 Movement Disorders Institute, Sheba Medical Centre, Ramat-Gan 5262000, Israel; haron.hassin@sheba.health.gov.il; 5 Department of Neurology, Sheba Medical Centre, Ramat-Gan 5262000, Israel; 6 Sackler Faculty of Medicine, Tel Aviv University, Tel Aviv 6997801, Israel

**Keywords:** assessment, activities of daily living, functional cognition, Parkinson’s disease

## Abstract

This study aimed to capture subjective daily functional cognitive decline among patients with Parkinson’s disease. Participants (40–79 y; 78 with Parkinson’s disease and 41 healthy matched controls) completed the Unified Parkinson’s Disease Rating Scale (MDS-UPDRS), Parkinson’s Disease Cognitive Functional Rating Scale (CFRS), Daily Living Questionnaire (DLQ), and Time Organisation and Participation Scale (TOPS) questionnaires. Patients with Parkinson’s disease were divided into groups with or without suspected mild cognitive decline according to their scores on the Cognitive Functional (CF) feature, which is based on certain items of the MDS-UPDRS. Significant between-group differences were found in the DLQ and TOPS scores. Significant correlations were found among the questionnaire results, with specific DLQ and TOPS items accounting for 35% of the variance in the CF feature, which correlated with daily cognitive functional states. This study’s results are relevant for detecting subtle deficits in Parkinson’s disease patients suspected of mild cognitive decline, which can affect health and quality of life and relates to risk for later dementia.

## 1. Introduction

The worldwide prevalence of Parkinson’s disease is increasing, and managing the condition is complex due to its progressivity, patient heterogeneity, wide range of symptoms, and increasing effect on functions in daily life [1]. About one-fourth of patients with Parkinson’s disease suffer from mild cognitive impairment (MCI). However, frequency rates of 9% to 65% of Parkinson’s disease cohorts have been reported in studies that applied Level I and/or II of the Movement Disorder Society (MDS) Parkinson’s disease MCI (PD-MCI) criteria [2,3], reflecting the difficulty in defining and identifying MCI. The PD-MCI represents a transitional phase between normal cognitive functioning and dementia, and is of utmost clinical relevance because it negatively affects patients’ quality of life [3]. Improved awareness and recognition of slight cognitive decline may enable early identification of PD-MCI and early symptomatic intervention to address patients’ difficulties and their caregivers’ concerns promptly. Thus, increasing knowledge about PD-MCI manifestations has therapeutic, epidemiologic, and prognostic implications [4].

In a previous study, we questioned the clinical application of the third diagnostic criteria for PD-MCI proposed by the MDS [5], indicating cognitive deficits on formal neuropsychological testing or a scale of global cognitive abilities. We found incompatibility between the Montreal Cognitive Assessment (MoCA) scores that serve as a global cognitive abilities scale (Level I criteria) and neuropsychological test scores (Level II criteria) among 78 Parkinson’s disease patients suspected of PD-MCI [6]. However, we found consistent results for participants’ subjective self-reports related to their cognitive functional abilities [6], as reflected by the Cognitive Failures Questionnaire [7] and the Parkinson’s Disease Cognitive Functional Rating Scale (PD-CFRS) [8]. Additionally, we found that self-reported perceived daily functional abilities validated by objective measures may contribute to the early identification of suspected PD-MCI [9]. These results led us to further study of ways to address the two diagnostic criteria for PD-MCI [5], which are based on the client’s or informant’s (family member or primary caregiver) subjective self-reports, or on what was observed by the clinician. These criteria still lack research and standardized, practical evaluation tools and are more complex to apply. They show the existence of a gradual decline in cognitive ability in the context of established Parkinson’s disease and criteria, and cognitive deficits that do not significantly interfere with functional independence, although subtle difficulties in complex functional tasks may be present [5]. The subjective impact of cognitive impairment on people with Parkinson’s disease has been found to be important in understanding the impact that individuals’ self-perceived cognitive impairments have on their everyday lives [10].

Notwithstanding the growing literature on PD-MCI, by 2022, its definition has not been further elucidated, nor has the impact of these cognitive impairments on patients’ daily functioning been delineated [11]. Questions still arise as to the extent of “gradual decline” and how to phrase questions to encourage patients to report such a decline and its impact. Practical tools to facilitate and enhance the accuracy of patients’ reports related to their cognitive functional abilities are scarce. The PD-CFRS [8] is one of two existing brief questionnaires developed for PD-MCI identification [3]. Finding additional specific tools is particularly important when considering the findings that PD-MCI may manifest diversely, reflecting the heterogeneity of the underlying pathology on the way to dementia [3]. Systematic identification of subtle deficits in functional abilities related to cognitive decline may benefit the detection and monitoring of PD-MCI, development of behavioral interventions, and perhaps slowing of functional deterioration [12].

In a previous article, we presented the idea of composing a Cognitive Functional (CF) feature as the mean score of seven items derived from the MDS’s Unified Parkinson’s Disease Rating Scale (MDS-UPDRS) [6,13]. We constructed this feature upon three concepts. First, Parkinson’s disease is not only a “movement disorder”, but also a “complex neurological disorder” with both motor and nonmotor manifestations [14]. Second, the ADL subscore of the MDS-UPDRS has previously shown a strong association with Parkinson’s disease duration, thus highlighting this score as an effective marker of the condition’s progression [15]. Lastly, according to the World Health Organization’s conceptual framework, normal functioning requires motor, cognitive, and psychological skills within appropriate environmental conditions [16,17]. Deficits in these skills may interact and contribute to impaired daily functioning. Accordingly, the CF feature created for this study combined seven items from the MDS-UPDRS Parts I and II. One item (1.1) is a classic nonmotor item assessing cognitive decline directly. Six items address motor experiences of daily living: 2.1 speech, 2.4 eating, 2.5 dressing, 2.6 hygiene, 2.7 handwriting, and 2.8 doing hobbies. Apart from motor functioning, these daily functioning tasks involve cognitive abilities such as attention, inhibition, and executive functions (e.g., planning, working memory, spatial orientation, and organization) [17,18,19]. The CF feature—but not the MoCA score—significantly correlated with self-report questionnaire scores reflecting daily cognitive functioning, including the PD-CFRS [6,8].

Consequently, for the present analysis, we divided the Parkinson’s disease patients into two groups based on their CF features. Our purpose was to determine whether this feature may serve as an indicator, differentiator, or predictor of functional cognitive decline as reflected by the daily living questionnaire (DLQ) [19] and the Time Organisation and Participation Scale (TOPS) [20]. Thus, we examined differences in functional cognition, as measured by subjective self-report questionnaires, between the two Parkinson’s disease patient groups based on their CF features and a healthy control group; assessed correlations among the PD-CFRS, MDS-UPDRS, DLQ, and TOPS scores in the entire Parkinson’s disease sample; and determined whether these scores predicted the CF feature.

## 2. Materials and Methods

### 2.1. Study Participants

This paper presents partial results of a larger study that included 119 participants who provided written informed consent [6]. Following institutional ethical approval from the Institutional Ethics Committee (protocol code 3852-17-SMC, 30 March 2017) and according to the Declaration of Helsinki, participants were invited for one or two study visits (maximum 2 weeks apart) at the Sheba Medical Centre. The study included patients diagnosed with probable or possible Parkinson’s disease according to the MDS clinical diagnostic criteria [21], with up to 10 y of Parkinson’s disease duration. A control group matched to the Parkinson’s disease group for gender, age, and education level was recruited via family members and friends of the Parkinson’s disease patients, the institute’s staff members, and social media.

Inclusion criteria for the Parkinson’s disease group and the control group were age 40 to 80 y; residence in Israel for at least 10 y; ability to speak, write, and read Hebrew; and normal or corrected vision and hearing ability. All participants were functionally independent, lived in private homes or assisted living facilities, and obtained scores of 18 or less on the Beck Depression Inventory (BDI) [22] and scores of 23 or more on the MoCA [23]. For the Parkinson’s disease group, patients were required to have a Hoehn and Yahr Stage 1 or 2 [24]. Exclusion criteria for the entire sample were a significant systemic disease (e.g., cancer or congestive heart failure), neurological condition (e.g., epilepsy or previous stroke, transient ischemic attack, brain tumor, surgery, or trauma), psychiatric disease (e.g., major depression, bipolar affective disorder, or obsessive-compulsive disorder), or treatment with antipsychotic or anticholinergic medications.

A movement disorders neurologist assessed Parkinson’s disease-related symptoms and signs in the Parkinson’s disease patients using patients’ self-reports, physical examinations, and the MDS-UPDRS [13]. The neurologist rated patients during their “on” state (i.e., a time when patients had taken their dopaminergic medication). Demographic and Parkinson’s disease-related clinical data, along with documentation of medical comorbidities and Parkinson’s disease medications, were collected from patient files. A total daily levodopa equivalent dose was calculated for each patient. The neurologist further evaluated patients for some exclusion criteria using the MDS-UPDRS depressed mood (1.3) and cognitive impairment (1.1) question scores, excluding those who scored 3 or more on either question. In addition to the MoCA, a battery of neuropsychological tests was administrated to all participants. Their results were described in the previous study [6].

In this study, the Parkinson’s disease patients were divided into two groups based on their CF features. The first group (score ≥ 1) contained patients with cognitive functional difficulties suspected of mild cognitive decline (s.MCD); the second group (score < 1) contained Parkinson’s disease patients (PD) without cognitive functional difficulties based on their reports [6].

### 2.2. Measures

A certified occupational therapist administered the self-report questionnaires, which focused on participants’ functional cognition.

#### 2.2.1. Parkinson’s Disease Cognitive Functional Rating Scale

The PD-CFRS is a reliable and valid 12-item self-report questionnaire designed to explore a range of functional aspects sensitive to early and mild cognitive decline in Parkinson’s disease relative to activities [8]. Scores range from 0 to 2; higher scores indicate higher decline. A Hebrew version was implemented in this study with the original author’s approval.

#### 2.2.2. Daily Living Questionnaire

The DLQ evaluates functional cognition through 52 items scored on a 5-point Likert scale [19]. For each item, respondents rate whether they have mental difficulty, scored as 0 (not applicable/not rated), 1 (no difficulty), 2 (some difficulty), 3 (a lot of difficulty), or 4 (unable to do). The 52 items are divided into two parts. Part A, Activities and Participation, includes the subscales of household tasks, activities involving language/comprehension, community/participation, and complex tasks. Part B, Cognitive Symptoms or Impairments, includes subscales of executive function, memory, and executive function monitoring. Internal reliability found in the current sample was α = 0.95 (DLQ A) and α = 0.96 (DLQ B).

#### 2.2.3. Time Organization and Participation Scale

The TOPS is a 34-item self-report standardized scale scored on a 5-point Likert-type scale [20]. It assesses respondents’ difficulties in time organization while performing daily tasks. It is divided into four parts, of which we used three (32 items): (A) pace of daily task performance (20 items) rated from 5 (always) to 1 (never); (B) way activities are organized throughout the day (five items) rated from 5 (excellent) to 1 (very bad); and (C) frequency of emotional responses following disorganization in time (seven items), rated from 5 (never) to 1 (always). Internal reliability in the current sample was α = 0.96, 0.91, and 0.85, respectively, for the TOPS A, B, and C.

### 2.3. Statistical Analysis

We conducted analyses using IBM SPSS software (version 23). Statistical assumptions for all analyses were tested with the significance set at *p* = 0.05. *T*-tests for continuous variables and chi-squared tests for categorical variables were conducted to identify significant between-group differences in demographic and medical status variables. We checked the internal variability of each self-report questionnaire’s domains using Cronbach’s alpha analysis. Because the self-report questionnaires were not normally distributed, nonparametric (Kruskal–Wallis *H* and Mann–Whitney *U*) tests were implemented to check for group differences across those measures. Further item-level comparison analysis of the functional cognition questionnaires enabled creation of mean scores of the DLQ and TOPS items that best differentiated the Parkinson’s disease patients with s.MCD from those without (PD group). Spearman correlations were conducted on the entire sample of Parkinson’s disease patients among the PD-CFRS, total, MDS-UPDRS parts, TOPS, and DLQ scores, including the computed TOPS and DLQ best scores. Stepwise regression was used to examine whether the TOPS best and DLQ best scores predicted the pre-established CF feature

## 3. Results

### 3.1. Participant Demographic Characteristics and Medical Status

Participants were 119 men and women aged 40 to 79 y (*M* = 63.62 y, *SD* = 8.52). Table 1 presents a comparison of the demographic characteristics of the three groups based on the CF feature: s.MCD group (*n* = 25, 17 men), PD group (*n* = 53, 37 men), and control group (*n* = 41, 23 men).

### 3.2. Correlations

Although significant group differences were found for age (Table 1), the Spearman analyses indicated no significant correlation between age and any of the PD-CFRS, TOPS, or DLQ questionnaire scores.

Significant differences were found in: (1) the MoCA scores between the PD and the control groups but not between the PD and s.MCD groups; (2) the BDI score between both the control and the PD groups and the s.MCD group; and (3) the CF feature between the PD and s.MCD groups based on their MDS-UPDRS scores, except for MDS-UPDRS Part IV (motor complications) and total daily levodopa equivalent dose.

As presented in Table 2, significant group differences were found between the PD and s.MCD groups for all DLQ and TOPS subscale scores except memory. When comparing the s.MCD and control groups for all DLQ and TOPS subscales, higher significant group differences were found. However, when comparing the PD and control groups, significant group differences were found only for the TOPS A (pace), DLQ A3 (community/participation), and DLQ B3 (monitoring) scores.

Table 3 presents the correlations between the PD-CFRS total, MDS-UPDRS Parts I to IV, and both functional cognition scale scores (DLQ and TOPS). Significantly high correlations were found between the PD-CFRS total score and the TOPS and DLQ domains for both the PD and s.MCD groups, as defined by the CF feature score. Moreover, significant correlations were found between the PD-CFRS score and the MDS-UPDRS Parts I (*r* = 0.422, *p* < 0.01) and II (*r* = −0.433, *p* < 0.01), but none with Parts III or IV.

Following the results given in Table 2 and Table 3, we carefully examined for TOPS and DLQ items that best differentiated between the two Parkinson’s disease groups (Table 4) and computed a mean score for those items on each scale.

Table 5 presents the results of stepwise regressions to determine whether the TOPS best scores and the DLQ best scores predicted the CF feature. The PD-CFRS score was not included in the regressions due to the highly significant correlations found in this score with the DLQ and TOPS scores (Table 3). The results in Table 5 indicated that age accounted for 10% of the variance of the CF feature, *F* (1,76) = 8.66, *p* = 0.004; whereas the DLQ best score added 29% to the prediction, *F* (2,75) = 36.27, *p* < 0.001; and the TOPS best score added 6%, *F* (3,74) = 7.94, *p* = 0.006. Together, the DLQ best and TOPS best scores accounted for 35% of the variance in the CF feature.

## 4. Discussion

This article presents a further effort to improve the identification of cognitive functional impairments in Parkinson’s disease and challenges the traditional concept that motor disability causes the most functional impairments [3]. Based on patients’ CF features, 25 of 78 (32%) patients were identified as s.MCD, a percentage similar to that previously described in the literature on PD-MCI [3,25].

Despite the similarity of the two Parkinson’s disease groups in most demographic and clinical features (i.e., disease duration, Hoehn and Yahr stage, MDS-UPDRS Part IV motor complications, and MoCA scores), the significantly higher MDS-UPDRS Parts I, II, and III scores found for the s.MCD group indicated that group’s impaired status. Their significantly higher depression may be explained by their decreased cognitive functional abilities in all types of daily tasks, including household, language/comprehension, community, and complex tasks reflected in the DLQ. Furthermore, the significant differences found for their executive function, memory, and executive function monitoring are supported by previous findings on the important contribution of executive and memory deficits for PD-MCI identification, whereas executive dysfunction has predicted the development of PD-MCI a few years after Parkinson’s disease diagnosis [26]. Moreover, deterioration in these cognitive components due to cortical and subcortical dysfunction that influence Parkinson’s disease patients’ ability to engage in basic and instrumental activities of daily living has been previously reported [3,27].

It is important to note that the DLQ scores in both Parkinson’s disease groups ranged from 1 (no difficulty) to 2 (some difficulty), meaning the people with suspected cognitive decline indeed feel subtle difficulties in their daily function [19], as expressed in the DLQ items. The s.MCD group also performed significantly lower in their TOPS organization-in-time skills [20]. However, their scores ranged from 3 to 4, meaning they seldom or only sometimes notice difficulties. These minimal changes in their daily function—which still significantly differed from those of the PD group—reflect their declines in cognition and daily living experiences. Such decline manifests in slow and subtle changes in the performance of daily tasks, including eating, speaking, and writing, but with no need yet for assistance. Because these tasks involve cognitive components such as focusing attention, inhibiting nonrelevant actions, planning action sequences, and considering space and time, slow performance may indicate decreased psychomotor speed accompanied by cognitive decline [28]. Thus, the small self-reported changes that occurred in performance abilities in daily tasks, as validated by the DLQ and the TOPS, may reflect a gradual cognitive decline as captured by the CF feature.

Significant correlations found in the current study between the MDS-UPDRS Parts I and II scores (but not Parts III and IV) and the Parkinson’s disease patients’ scores on the cognitive functional questionnaires (PD-CFRS, DLQ, and TOPS) indicated consistency in participants’ subjective reports when participants were asked appropriate questions related to real-life daily task-performance characteristics. Such questions enable Parkinson’s disease patients to reflect on being in the “preclinical” transitional stage between functional impairment and disability diagnosis that is characterized by a decline in the ability to perform physical tasks [29]. De Vriendt et al. described this stage as the process of functional decline in which adaptation and coping mechanisms interact with the process of reduced skills, leading to activity disruption and insufficient functioning [16]. This functional decline is supported by findings that underpin the role of patients’ ADL functioning in identifying functional decline in PD [9,15].

A picture of the patients’ daily life stories may be clarified by analyzing the specific DLQ best and TOPS best items that significantly differentiated the Parkinson’s disease groups. These items reflect the challenges and the lengthy time spent performing daily activities, such as planning and preparing meals, following conversations, participating in group discussions, or understanding new information. Such difficulties in cognitively demanding daily tasks align with previous findings among patients with PD-MCI [30] but are reflected in more basic tasks (cognitive function) that are less cognitively demanding [31]. Such difficulties reduce engagement in social interactions and can cause more social isolation, leading to reduced physical and mental health and motivation [26]. The implication of these functional difficulties in daily life possibly explains the higher depression scores (BDI) seen in the s.MCD group.

Finally, when grouped by the differentiating items, the DLQ and TOPS best scores indeed predicted the CF feature. Whereas some previous research found that most PD-MCI patients displayed deficits in a single cognitive domain [32], others identified multiple-domain impairments as more frequent [33]. However, individual daily life activities are complex and require integrative use of more than a single cognitive or executive function domain. The functional deficits revealed in the self-report questionnaires reflect the complexity of such daily activities. Underscoring the complexity of daily activities aligns with the MDS diagnostic criteria for PD-MCI, which state that daily life encounters involve performing complex daily tasks [30].

## 5. Limitations

Some limitations of the current study cannot be ignored. Participants in this study’s sample had high education levels, which could influence some participants’ ability to respond to the self-report questionnaires, but may not reflect all informants. In addition, the sample’s maximum Hoehn and Yahr score was 2.3; thus, it may not represent the full range of disability levels. Finally, the results were based on subjective yet standardized self-report questionnaires. Further studies are required to analyze the relationships between subjective and objective measures of cognitive decline among Parkinson’s disease patients. In addition, future research that investigates cognitive deterioration in the context of overlaps in the early stages between Parkinson’s disease and Progressive Supranuclear Palsy-Parkinsonism Predominant (PSP-P) [34,35] is required.

## 6. Conclusions

Our results indicated that the CF feature supports gradual cognitive decline detection based on seven simple self-reported daily functioning items already routinely collected [6]. Furthermore, it can be validated by the DLQ and TOPS best items: a short, practical scale. Combining the CF feature with the DLQ and TOPS best items enables early identification of subtle cognitive deficits. Such identification can prevent emotional and physical health consequences in functionality and maintain and improve quality of life [36,37].

Comprehensive cognitive evaluations are costly, time and energy consuming, and, as these and previous results showed, may not fully reflect preserved or affected individuals’ perceived functional cognitive abilities as PD-MCI markers [5,6,14]. The CF feature was derived for the current study from an established scale that is extensively and routinely used [1]. This suggested feature may be beneficial for clinical use if the MDS provides permission to use it; it may serve as a simple and quick initial checklist to reveal early signs of MCI reflected in real-world functional cognitive decline. Identifying a daily life story can direct the administration of additional functional cognitive assessments to confirm the fourth MDS criterion [5], assist caregivers and clinicians in providing care in these areas, and lead to improved health and quality of life for Parkinson’s disease patients.

## Figures and Tables

**Table 1:** Comparison of group demographic characteristics and medical status of the PD patients with (s.MCD group) and without (PD group) mild cognitive impairment based on their CF feature and healthy controls.

Variable	Group				
	s.MCD (*n* = 25)	PD (*n* = 53)	Control (*n* = 41)		
	%			*F*, *t, χ^2^*	*p*
Gender (% men)	68.00	69.80	56.00	χ^2^ = 2.030	0.360
Hand dominance (% right)	88.00	90.60	90.20	χ^2^ = 3.190	0.520
Country of birth (% Israel)	84.00	75.50	68.30	χ^2^ = 14.720	0.140
Patients treated with levodopa (%)	48.00	49.10		χ^2^ = 0.008	0.560
	*M (SD)*				
Education (y) *	16.40 (3.68)	15.64 (3.67)	16.88 (3.57)	*F* (2, 116) = 3.100	0.049
Age (y), *M* (*SD*)	60.24 (8.36)	66.26 (7.24) ^a^ *	62.27 (9.26)	*F* (2, 116) = 5.400	0.006
MoCA score	26.00 (2.00)	24.98 (2.00)	26.05 (1.56) ^c^ *	*F* (2, 116) = 4.530	0.013
BDI score	7.79 (4.92)	5.43 (4.43) ^a^ *	3.18 (3.66) ^b^ ***, ^c^ *	*F* (2, 111) = 8.750	<0.001
PD duration since diagnosis (y) *	4.09 (2.88)	4.21 (3.42)		*t* (76) = −0.141	0.880
MDS-UPDRS P-I *	9.16 (6.49)	0.5 (3.32)		*t* (76) = 3.750	<0.001
MDS-UPDRS P-II *	15.52 (4.52)	6.13 (3.02)		*t* (76) = 10.850	<0.001
MDS-UPDRS P-III *	32.4 (12.66)	23.23 (10.09)		*t* (76) = 3.460	0.001
MDS-UPDRS P-IV *	2.12 (3.37)	1.38 (3.12)		*t* (76) = 0.940	0.350
MDS-UPDRS total	59.24 (17.83)	35.77 (13.62)		*t* (76) = 6.450	<0.001
LED (Mg) *	509.34 (400.07)	316.8 (297.05)		*t* (76) = −2.380	0.020
Hoehn and Yahr stage “on” *	Range: 1.0–2.5; Median: 2	Range: 1.0–3.0; Median: 2			

Legend: PD = Parkinson’s disease; s.MCD, suspected mild cognitive decline; BDI, Beck Depression Inventory; MDS-UPDRS P-I, nonmotor experiences of daily living; MDS-UPDRS P-II, motor experiences of daily living; MDS-UPDRS P-III, motor examination; MDS-UPDRS P-IV, motor complications; MoCA, Montreal Cognitive Assessment; LED, total daily levodopa equivalent dose; Mg, milligrams. ^a^ Significant differences between suspected mild cognitive decline and PD; ^b^ between suspected mild cognitive decline and control; ^c^ between PD and control. * *p* < 0.05, *** *p* ≤ 0.001.

**Table 2:** Comparison of the DLQ and TOPS scores of the PD patients with (s.MCD group) and without (PD group) suspected mild cognitive decline based on their CF features and healthy control groups.

Variable	Group *M* (*SD*), Mean Rank			*χ2(2)*	*p*
	s.MCD (*n* = 25)	PD (*n* = 53)	Control (*n* = 41)		
DLQ					
DLQ A: Activities and participation	1.56 (0.49), 82.00	1.24 (0.27) ^a^ *, 57.75	1.17 (0.20) ^b^ **, 49.50	14.27	0.001
1. Household tasks	1.48 (0.56), 78.02	1.17 (0.23) ^a^ *, 57.66	1.12 (0.19) ^b^ ***, 52.04	10.32	0.006
2. Language/comprehension	1.66 (0.55), 81.48	1.27 (0.35) ^a^ *, 56.95	1.21 (0.32) ^b^ ***, 50.84	14.01	0.001
3. Community/participation	1.51 (0.50), 80.04	1.23 (0.36) ^a^ *, 60.25	1.09 (0.16) ^b^ ***, 47.46 ^c^ *	16.06	<0.001
4. Complex tasks	1.58 (0.54), 75.58	1.33 (0.40) ^a^ *, 56.47	1.31 (0.36) ^b^ *, 55.06	6.78	0.034
DLQ B: Cognitive symptoms/impairments	1.74 (0.59), 81.30	1.35 (0.32) ^a^ *, 58.77	1.25 (0.25) ^b^ ***, 48.60	14.13	0.001
1. Executive functions (EF)	1.79 (0.62), 80.06	1.37 (0.35) ^a^ *, 57.48	1.31 (0.33) ^b^ **, 51.02	11.68	0.003
2. Memory	1.62 (0.56), 75.34	1.35 (0.38), 60.57	1.21 (0.24) ^b^ *, 49.91	9.15	0.010
3. EF Monitoring	1.75 (0.62), 83.88	1.31 (0.35) ^a^ *, 59.49	1.18 (0.26) ^b^ ***, 46.10 ^c^ *	19.37	<0.001
TOPS					
TOPS A: Pace of daily task	3.87 (0.73), 34.18	4.40 (0.58) ^a^ *, 59.38	4.69 (0.28) ^b^ ***, 76.55 ^c^ *	23.53	<0.001
TOPS B: How activities are organized	3.44 (0.67), 34.06	4.01 (0.79) ^a^ *, 61.72	4.29 (0.63) ^b^ **, 73.60	20.91	<0.001
TOPS C: Emotional responses	3.78 (0.68), 41.62	4.17 (0.57) ^a^ *, 59.71	4.37 (0.44) ^b^ *, 71.59	11.79	0.003

Legend: PD, Parkinson’s disease; PD-MCI, PD mild cognitive impairment; PD-CFRS, Parkinson’s Disease Cognitive Functioning Rating Scale; DLQ, Daily Living Questionnaire; TOPS, Time Organisation and Participation Scale. PD-CFRS < better performance, TOPS > better performance, DLQ < better performance. ^a^ Significant differences between suspected mild cognitive decline and PD; ^b^ between suspected mild cognitive decline and control; ^c^ between PD and control. Effect size (r) = 0.1, small; 0.3, medium; 0.5, large. * *p* < 0.05, ** *p* ≤ 0.01, *** *p* ≤ 0.001.

**Table 3:** Nonparametric correlations between the PD-CFRS total score, the MDS-UPDRS subscales and total scores, and the TOPS and DLQ domains in the sample of Parkinson’s disease patients (*n* = 78).

Domain	PD-CFRS Total Score	MDS-UPDRS Part I	MDS-UPDRS Part II	MDS-UPDRS Part III	MDS-UPDRS Part IV
DLQ A: Activities and participation	0.606 ***	0.524 ***	0.474 ***	0.032	−0.052
1. Household tasks	0.570 ***	0.408 ***	0.328 **	−0.105	0.068
2. Language/comprehension	0.581 ***	0.425 **	0.400 ***	0.020	−0.134
3. Community/participation	0.477 ***	0.497 ***	0.440 **	0.155	−0.098
4. Complex tasks	0.494 ***	0.491 **	0.426 **	0.060	−0.156
DLQ B: Cognitive symptoms/impairments	0.605 ***	0.536 ***	0.444 **	0.172	−0.081
1. Executive functions	0.608 ***	0.560 **	0.459 ***	0.188	−0.098
2. Memory	0.467 ***	0.361 **	0.276 **	0.034	−0.060
3. Monitoring	0.613 ***	0.484 **	0.453 **	0.201	−0.055
DLQ best score	0.606 ***	0.563 ***	0.515 ***	0.197	−0.064
TOPS A: Pace of daily task performance	−0.460 ***	−0.396 ***	−0.496 ***	−0.194	0.234
TOPS B: How activities are organized	−0.362 ***	−0.404 ***	−0.395 ***	−0.031	−0.010
TOPS C: Emotional response	−0.536 ***	−0.351 ***	−0.257 **	0.131	−0.019
TOPS best score	−0.513 ***	−0.475 ***	−0.551 ***	−0.171	−0.111

Legend. PD-CFRS, Parkinson’s Disease Cognitive Functioning Rating Scale; MDS-UPDRS, Movement Disorder Society’s Unified Parkinson’s Disease Rating Scale, Part I (nonmotor experiences of daily living), Part II (motor experiences of daily living), Part III (motor examination), Part IV (motor complications); TOPS, Time Organisation and Participation Scale; DLQ, Daily Living Questionnaire. ** *p* < 0.05, *** *p* < 0.01.

**Table 4:** Differences in TOPS and DLQ items with significance level of *p* ≤ 0.01 between the Parkinson’s disease patients with and without suspected mild cognitive decline based on their cognitive functional feature.

Item Number	Item Description
	DLQ
4	Planning and preparing meals
6	Household tasks (organizing laundry)
10	Planning/choosing what to wear
22	Planning social arrangements with family, friends or for children
35	Following a conversation
36	Participating in group discussions
41	Understanding new information
46	Handling complex tasks that include keeping track of a lot of information at once
54	Planning and thinking ahead
58	Switching easily from one task to another
61	Managing multiple step tasks
64	Accomplishing tasks within a reasonable timeframe
65	Responding quickly to situations when necessary
69	Taking initiative to start a new activity or project
	TOPS
A2	Toileting
A3	Washing face, brushing teeth, combing hair
A4	Getting dressed
A6	Bathing/taking a shower
A11	Fulfilling varied roles
A12	Completing tasks/work that you take upon yourself
A17	Planning leisure activities
A20	Carrying out varied activities required at work or at school
B4	Organizing your time in preparation for studying or work
B5	Performing your everyday tasks at an appropriate pace
C1	Lack of motivation to perform

Legend: DLQ, Daily Living Questionnaire; TOPS, Time Organisation and Participation Scale.

**Table 5:** Stepwise regression of prediction of the MDS-UPDRS cognitive function (CF) feature by age and functional cognition scales (DLQ and TOPS).

Variable	Model 1			Model 2			Model 3		
	B	*SE* B	β	B	*SE* B	β	B	*SE* B	β
Age	−0.021	0.007	−0.320 *	−0.017	0.006	−0.250 *	−0.016	0.006	−0.238 *
DLQ Best score				0.677	0.112	0.545 ***	0.458	0.133	0.368 ***
TOPS Best score							−0.250	0.089	−301 ***
*R* ^2^	10			39			45		
*F* change *R*^2^	8.660 *			36.270 ***			7.940 *		

Legend: MDS-UPDRS, Movement Disorder Society’s Unified Parkinson’s Disease Rating Scale; DLQ, Daily Living Questionnaire; TOPS, Time Organisation and Participation Scale. * *p* < 0.05, *** *p* ≤ 0.001.

## Data Availability

The data presented in this study are available upon request from the corresponding author. The data are not publicly available due to the privacy of the participants.
